# Electrospinning technology: a promising approach for tendon–bone interface tissue engineering

**DOI:** 10.1039/d4ra04043k

**Published:** 2024-08-19

**Authors:** Chengzhi Liang, Zaiwei Fan, Zirui Zhang, Pinkai Wang, Hui Deng, Jun Tao

**Affiliations:** a Department of Orthopaedics, The Second Affiliated Hospital of Nanchang University Nanchang Jiangxi 330000 China ndefy14038@ncu.edu.cn; b Department of Rehabilitation Medicine, The 960th Hospital of the Chinese People's Liberation Army Jinan 250000 China

## Abstract

The regeneration of tendon–bone interface tissue has become a topic of great interest in recent years. However, the complex nature of this interface has posed challenges in finding suitable solutions. Tissue engineering, with its potential to improve clinical outcomes and play a crucial role in musculoskeletal function, has been increasingly explored for tendon–bone interface regeneration. This review focuses on the research advancements of electrospinning technology in interface tissue engineering. By utilizing electrospinning, researchers have been able to fabricate scaffolds with tailored properties to promote the regeneration and integration of tendon and bone tissues. The review discusses the unique structure and function of the tendon–bone interface, the mechanisms involved in its healing, and the limitations currently faced in achieving successful regeneration. Additionally, it highlights the potential of electrospinning technology in scaffold fabrication and its role in facilitating the development of functional and integrated tendon–bone interface tissues. Overall, this review provides valuable insights into the application of electrospinning technology for tendon–bone interface tissue engineering, emphasizing its significance in addressing the challenges associated with regeneration in this complex interface.

## Abstract 2

This review highlights recent advances in tendon–bone interface (TBI) tissue regeneration, focusing on the application of electrospinning technology.

## Graphical abstract

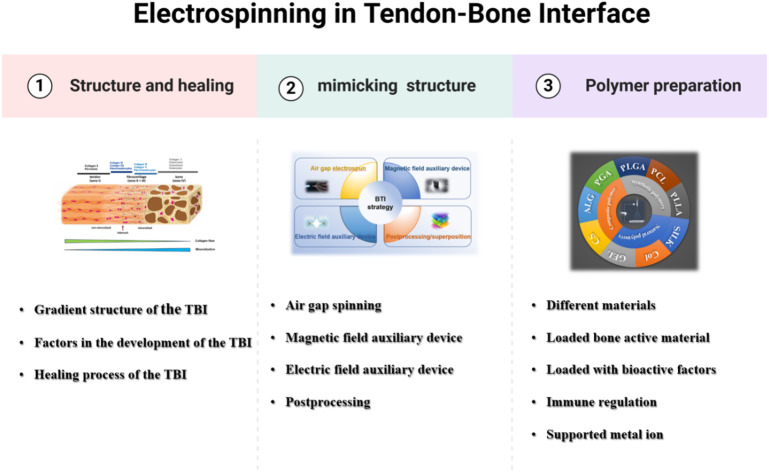

## Introduction

The musculoskeletal system of the human body functions through the coordinated actions of various tissues, providing support and stability while allowing organized movement of muscles and bones. Ligaments attach bones to bones and tendons connect muscles to bones, making connective tissue an important part of our bodies. The transitional portion from muscle to bone, known as the tendon–bone interface (TBI), is a highly specialized site that can effectively transmit tensile loads from soft to hard tissues.^1^ These interfaces demonstrate gradient changes in structural, compositional and mechanical properties to efficiently transmit stresses between tendons and bones.^2,3^ Tears of tendon or ligament insertions are common clinical problems encountered in orthopedic practice. Injuries to the rotator cuff are among the most common sports injuries, usually causing pain, weakness, and limited range of motion in the shoulder, ultimately imposing a heavy financial burden on families and society.^4^ Rotator cuff tears restrict shoulder joint movement and severely impact patients' daily lives. Surgical repair is usually needed for rotator cuff tears. While some patients see significant improvements in shoulder function post-surgery, rerupture rates are still high, ranging from 15–94%.^5,6^ The high rerupture rate can be attributed to the fact that the injured site is typically located at the tendon–bone interface, a complex structure and composition that makes repair difficult, while scarring causes weak tissues.^7,8^ Faced with the current situation, rotator cuff surgery remains a challenge and better solutions need to be developed to avoid postoperative reruptures.

**Fig. 1:**
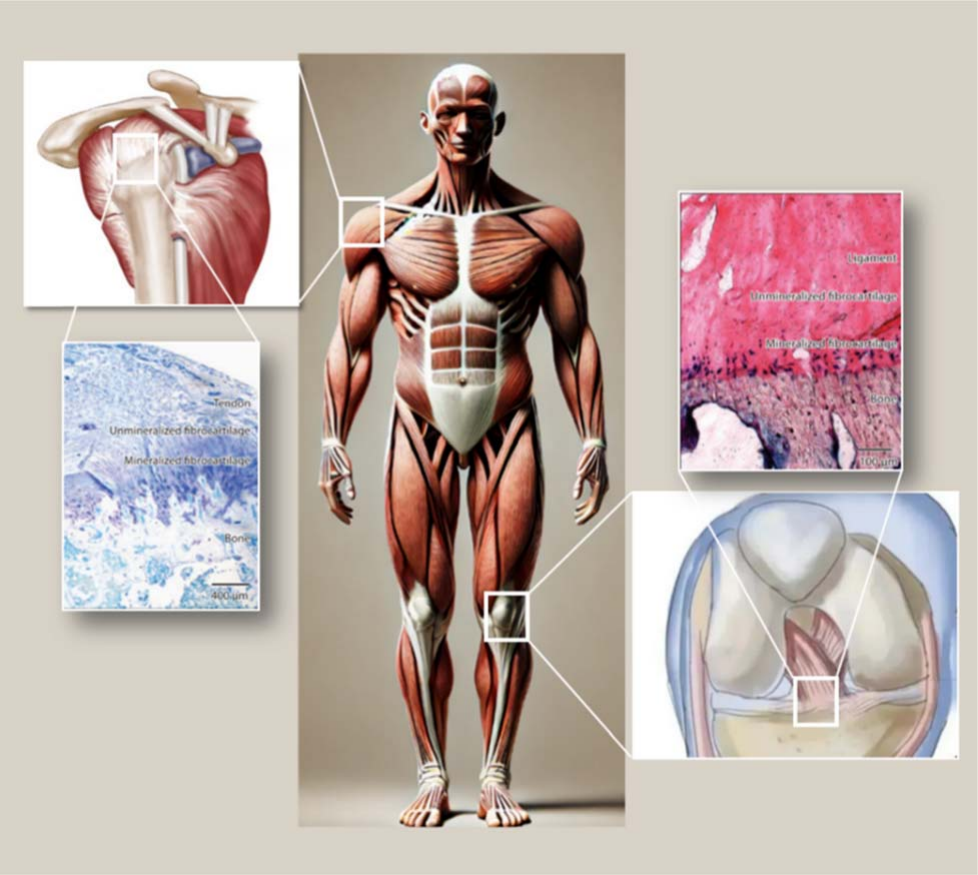
Common anatomical locations of fibrocartilage insertions in the human body and a schematic diagram illustrating the transitional zones within the insertion site.^10^

Between tendons and bones, there are four layers of structural and compositional transition: tendon, non-mineralized fibrocartilage, mineralized fibrocartilage, and bone^9^ (Fig. 1). Within the tendon–bone interface, cells and extracellular matrix are arranged in a gradient direction along the interface, with this transitional zone mediating load transfer from tendon to bone and helping transmit forces from the relatively soft tendon tissue to the rigid bone, so as to minimize stress concentrations. However, in the healing process from tendon to bone, this unique transitional tissue between tendon and bone is not reconstructed.^5^ There may be issues with fibrocartilage regeneration, bone loss, and immune dysregulation due to an imbalance between pro-inflammatory and anti-inflammatory macrophages after injury at the bone–tendon interface.^11,12^ During the early stages of rotator cuff tears (RCT), pro-inflammatory macrophages disproportionately produce interleukin 1 (IL-1), interleukin 6 (IL-6), and tumor necrosis factor (TNF-α),^13^ excessive secretion of inflammatory factors inhibits fibrocartilage layer regeneration and enhances osteoclast activity.^12^ As a result, M2 macrophages do not have sufficient numbers to support bone and fibrocartilage regeneration during tendon–bone healing.^12^ Therefore, it is feasible to improve the abnormal inflammatory response, promote cartilage regeneration, reduce bone loss and promote osteogenic differentiation in the process of tendon–bone healing.^14,15^

In recent years, tissue engineering of the tendon–bone interface has gained increasing attention as a potential therapeutic option. For tissue repair, tissue engineering is important because it can mimic natural tissues and provide an extracellular matrix (ECM) environment that mimics natural tissues. ECM microenvironment regulates stem cell behaviors and fates in important ways. With electrospinning, polymer fibers can be produced with diameters of 50–1000 nm, which are several orders of magnitude smaller than fibers produced with other conventional fabrication methods.^16^ It has therefore been proposed that electrospinning can produce scaffolds composed of fibers that are more similar to natural collagen fibers in tendons in terms of their diameter scale and layered structures. There have been numerous studies showing that electrospun nanofiber scaffolds promote cellular adhesion, growth, proliferation and even differentiation in tendon tissue engineering applications, as well as demonstrating promising regenerative outcomes.^17^

This review focuses on the tendon–bone interface tissue. First, the current development characteristics of the attachment points of bone tendon and the healing mechanism of the injured interface are discussed. In the following sections, electrospinning technology will be primarily discussed in the context of tendon and bone interface tissue engineering. The second part introduces the design strategy of biomimetic stents prepared by loading active substances, last but not least, it summarizes the field's potential challenges, as well as future directions.

## The structural and development of the tendon–bone interface

**Fig. 2:**
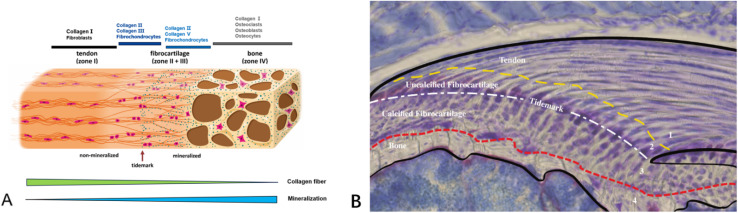
(A) Schematic diagram illustrating the structural and compositional changes within the transitional zones at the tendon–bone insertion site.^21^ (B) Schematic diagram of an histopathological section of the supraspinatus tendon–bone insertion in mice. The Alcian blue staining of proteoglycans in tendon, fibrocartilage, and mineralized fibrocartilage shows the presence of proteoglycans, highlighting the compositional gradient characteristic of the insertion site.^10^

Tendon–bone transitions *in vivo* occur in two forms – fibrous attachments and fibrocartilaginous attachments.^18^ The characteristic of fibrous attachments is that the tendon–bone interface has dense fibrous connective tissue, found in tendons and ligaments attaching to the shaft or end of long bones (such as the medial collateral ligament, triceps tendon).^19,20^ Fibrous attachments can be further divided into two types: entheses and osteotendinous junctions. In the former, the tendon is indirectly attached to the bone by the periosteum, while in the latter, it is directly attached to the bone by the tendon.^19^ In comparison, fibrocartilaginous attachments occurring at bone protuberances and epicondyles (including the rotator cuff and anterior cruciate ligament) (Fig. 1) are more commonly encountered in human injuries.^19,20^ This paper will discuss fibrocartilaginous attachments. Fibrocartilage at bone attachments can be divided into four transitional zones (Fig. 2). A transition occurs between soft tissues and hard tissues at these four areas, which contain different collagen, minerals, cells, and other substances. The aim of this transition is to transmit external loads between soft tissues and hard tissues, ensuring stresses are minimized as much as possible and promoting joint movement. Zone I: fibrious connective tissue zone with elongated fibroblasts and mainly type I collagen, dispersed in a polysaccharide and glycoprotein matrix, with little type III collagen and elastin.^10,22–24^ Zone II: unmineralized fibrocartilage zone, avascular and mainly composed of type II and III collagen forming a mesh-like structure. This region also contains small amounts of type I collagen, aggrecan proteoglycans, cartilage-specific chondroitin sulfate glycosaminoglycans (GAGs).^9,20–22,25–27^ It delineates zones II and III mechanically by following unmineralized fibrocartilage and is called the tidemark.^22^ Zone III: mineralized fibrocartilage zone, avascular and principally comprised of type II collagen and enlarged fibrocartilage cells surrounded by proteoglycans, type I and X collagens. This region is highly irregular and represents the true junction between tendon and bone, interfacing with subchondral bone.^21,22,27^ Zone IV: an osteoclastic, osteocyte, and osteoblastic zone of bone tissue that contains mineralized type I collagen.^9,22,27,28^ While these four tissue regions differ compositionally, structurally they are continuous. There is a gradual increase in mineral content and decrease in collagenous fiber organization during the transition from tendon into bone. The fibers are also aligned and parallel at the tendon origin but become more curved, crossed and disorganized nearer the bone.^9^ This architecture provides a more rational stress distribution, enhances adherence strength and reduces risk of rupture or tearing^24^ (Table 1).

**Table 1:** Zone of fibrocartilaginous enheses

	Zone 1 (dense fibrous connective tissue)	Zone 2 (uncalcified fibrocartilage)	Tidemark	Zone 3 (calcified fibrocartilage)	Zone 4 (bone)
Composition	Fibroblasts	Fibrochondrocytes		Fibrochondrocytes	Osteocytes
	Type I collagen	Proteoglycan aggrecan		Type II collagen	Osteoblasts
	Type III collagen	Collagen (types I–III)		Type I collagen	Osteoclasts
				Type X collagen	Type I collagen
Significance	Linearly arranged collagens whose mechanical properties are similar to that of the mid-substance tendon	Dissipates bending of collagen fibers in tendon	The basophilic demarcation between uncalcified and calcified fibrocartilage, representing the boundary between soft and hard tissues	Irregularity of attachments into bone give mechanical integrity of enthesis	Provides an attachment site for tendons

While new tendons and bones emerge almost simultaneously during fetal development, formation of the transitional tissue between them occurs postnatally.^29–34^ In the initial stages of development, cartilage is mineralized to form bone through endochondral ossification, followed by fibrocartilage transition at the interface. During embryonic skeletal development, progenitor cells in the primary cartilage template promote bone protuberances to serve as tendon anchor points.^35–37^ During tendon formation and differentiation, Scleraxis (Scx) is an important transcription factor. The Scleraxis gene is found in progenitor cells as well as in cells of all tendinous tissues.^38–40^ Experiments show Scx knockout leads to severe developmental abnormalities of insertions.^35^ It plays an important role in chondrogenesis through transcription, expression in proliferating chondrocytes, and differentiation of chondrocytes.^41,42^ SOX9 remains active throughout early and late stages of chondrogenesis. With advancing development and differentiation, expressions of SOX9 and SCX gradually decrease regulated by various molecular drivers, including the transforming growth factor β (TGF-β) subfamily as a major factor since TGF-β signaling controls progenitor cell behavior and is crucial for tendon and cartilage formation.^43^ TGF-β also modulates SCX expression, with lack of SCX leading to abnormal tendon and ligament development.^44^ In addition to TGF-β, bone morphogenetic protein (BMP) is another influential factor. BMP-4 colocalizes with SCX in progenitor cells of bone protuberances and is regulated by SCX, inducing protuberances formation in the tendon–cartilage attachment region. The SCX/BMP-4 signaling transduction is necessary for these progenitor cells to differentiate into cartilage without the SCX/BMP-4 signaling transduction.^29,30^ It is thought that Indian hedgehog (Ihh) and parathyroid hormone-related protein (PTHrP) promote chondrocyte proliferation and differentiation, which act as a negative feedback loop in order to maintain the number and quality of chondrocytes.^21^ Research elucidated extracellular matrix expression during development with fibroblasts characteristically expressing type I collagen, collagen type II chondrocytes and collagen type X chondrocytes.^30,31^

## Current understanding of tendon–bone interface healing

Current animal studies have shown that, unlike the organized and distinct development of four regions with cartilage attachment, in the case of tendon healing, scar tissue forms over the natural site of insertion, rather than reconstructing the site of insertion during embryonic development.^20,22,45–49^ The development of this fibrovascular scar tissue occurs in three stages: inflammation (0–7 days), repair (5–14 days), and remodeling (>14 days).^50,51^ The inflammation stage begins with platelet deposition of fibrin and fibronectin, leading to macrophage response and accumulation of insulin-like growth factor 1 (IGF-1), platelet-derived growth factor (PDGF), and transforming growth factor β (TGF-β).^22^ Getting tendon attachments healed requires TGF-1 and TGF-3, which are responsible for growth and differentiation of skeletal muscle.^52,53^ A significant amount of TGF-1 is responsible for cell migration and angiogenesis, while TGF-3 has been found to have an important role in the regeneration of articular cartilage for adults and in the healing of scarless wounds in newborns.^54–56^ When macrophages begin to secrete TGF-β1, the transition to the repair stage occurs, leading to fibroblast proliferation and scar tissue formation. Type III collagen is the main component of scar tissue.^55^ Although some degree of healing is achieved when tendon tissue is surgically fixed to bone, physiologically normal tissues have different mechanical properties from repaired tissues,^57^ even differing by orders of magnitude.^57^ Histologically, there are significant differences between the healed tissue and the physiological state, with loss of continuity in collagen fibers and no apparent gradient in mineral content, and the tissue at the healing interface consists of disorganized scar tissue.^58^ Damage to the interface between soft tissue and bone is accompanied by bone loss,^58,59^ making the repair of soft tissue to bone more complex. The bone mineral density of rat rotator cuffs was significantly reduced after tendon injury and repair.^60^ Similar results were found in a study on canine distal phalanges, with decreased bone mineral density suggesting that bone resorption may be a contributing factor to the poor outcomes at the repair site.^59^

In the development of tendons and bones, mechanical loading plays a significant role.^61^ Although the role of mechanobiology in the healing process is not yet clear, all cell types found near the attachment site have shown mechanical responsiveness.^62^ Current research suggests that cells are able to convert mechanical signals into gene regulation, which not only affects cell migration but also proliferation and differentiation.^63^ Additionally, the differences in the quantity of fibrocartilage found in different attachment sites may be related to mechanical forces.^64,65^ Muscle loading is essential for the growth and maturation of the attachment site, as evidenced by the reduction in mineral deposition and fibrocartilage formation observed when muscle loading is reduced using botulinum toxin, which affects postnatal attachment site maturation,^66^ resulting in unorganized fiber distribution and poorer mechanical properties.^67^ Several animal models have yielded similar results, with low-level loading (*e.g.*, plaster fixation) being optimal for healing.^57,68,69^ A rotator cuff injury animal model showed that plaster fixation was more effective than exercise at promoting tendon healing.^57,68^ Similar results were found in a study comparing the effects of immediate and delayed mechanical loading on tendon–bone healing in an ACL model. Delayed loading led to greater healing than immediate or prolonged loading.^57,69^ In addition to mechanical stimuli, other physical factors such as electrical stimulation, ultraviolet radiation, and sound waves (*e.g.*, ultrasound) have been studied. Through the stimulation of biological cascades, the increase of growth factors and cytokines levels, and the regulation of gene expression, they promote cell proliferation, differentiation, and osteogenesis. For example, low-intensity pulsed ultrasound (LIPUS) has been shown to increase levels of vascular endothelial growth factor (VEGF), thereby significantly improving vascular distribution at the attachment site.^70,71^

Cell phenotype and intercellular communication are another important influencing factor in the regeneration of the attachment site. During the insertion of tendon into bone, tendons, fibrocartilages, mineralized fibrocartilages, and bone are present. Each tissue type exhibits its own cell phenotypes and matrix composition. Interactions between the three resident cell populations can be crucial for fibroblast, fibrochondrocyte, and osteoblast regeneration at the interface. Osteoblast–fibroblast interactions mediated by heterotypic cell interactions can contribute to attachment site regeneration, resulting in osteoblastic and/or fibroblastic transdifferentiation. The interactions may also lead to the differentiation of stem cells into fibrocartilage cells, thereby regenerating the interface between the cartilage and the fibrocartilage.^72^ Numerous *in vitro* studies have also demonstrated the importance of heterotypic cell interactions in enhancing regeneration at interfaces.^73–75^*In vitro* studies were conducted using co-cultures and tri-cultures using interface-related cell populations to examine the influence of cell communication on the development of fibrocartilage-specific markers.^76–78^ It is still unclear how intercellular communication works and what modulatory soluble factors are secreted in the healing environment, but intercellular communication encourages interface regeneration in both homing and healing environments.

## Strategies for electrospinning to reconstruct special structures

**Fig. 3:**
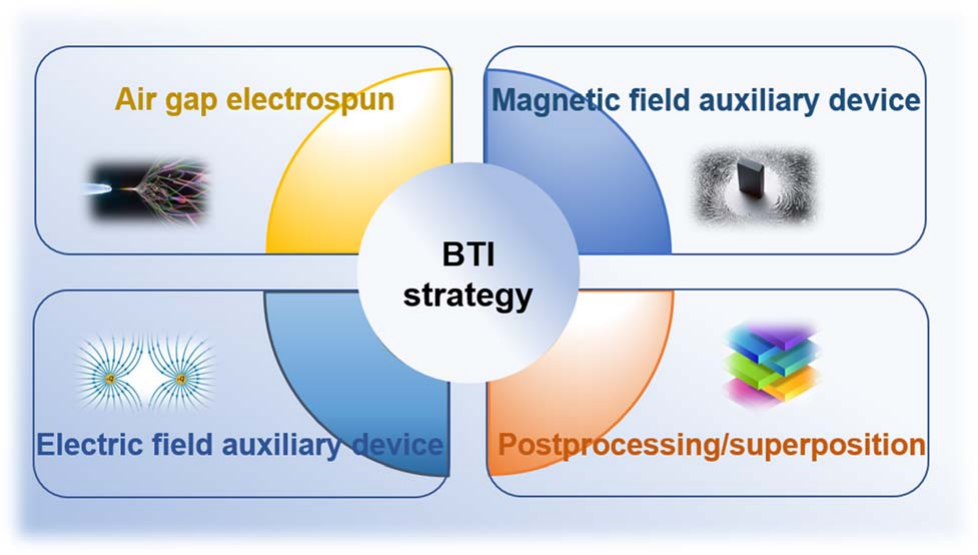
Schematic representation of manufacturing strategies for biomimetic scaffolds in interface tissue engineering.

Due to their high surface-to-volume ratios and interconnected porosities, fiberous materials have attracted a lot of attention from tissue engineering researchers, and diverse characteristics. Currently, there are several methods available for synthesizing nanofiber networks, including phase separation, template synthesis, and self-assembly.^79,80^ However, these methods have drawbacks such as long preparation time, limited fiber length, and discontinuous fiber scaffold structure.^81^ Therefore, electrospinning technology stands out among many nanofiber fabrication methods due to its obvious advantages, including the ability to produce continuous fiber networks relatively quickly and the versatility to work with various materials. Using a strong electric field, electrospinning produces micro- and nanofibers by jetting spinning from polymer melts or solutions. It is a novel processing method for producing nanoscale ultrafine fibers and can rapidly and cost-effectively manufacture nonwoven fiber structures.^82,83^ This fiber formation technique overcomes surface tension of the solution by applying voltage, promoting the formation of Taylor cones, jetting streams that pass through the air, and stretching out fibers with diameters ranging from nanometers to micrometers. These fibers deposit on collectors with opposite charges or grounded.^84^ Currently, most tissue engineering scaffolds produced are relatively homogeneous and possess isotropic mechanical properties. One end of a tendon–bone repair scaffold should have uniaxially aligned nanofibers, while the other end should have randomly oriented nanofibers.^16,17^ Additionally, it should mimic the mineralization changes at the tendon–bone interface, with a structure that gradually increases in hardness from soft to hard. It has been reported that electrospun polymer scaffolds with aligned fibers can enhance stem cell differentiation, alignment, and collagen fiber formation,^85–90^ while randomly oriented fibers play a role in osteogenesis and chondrogenesis.^91^ Although scaffolds with compositional, structural, and mechanical property gradients may have the potential to promote tendon–bone interface tissue growth, it is challenging to manufacture structures that transition from aligned fibers to randomly oriented fibers. Therefore, new manufacturing strategies are needed to prepare biomimetic scaffolds for interface tissue engineering (Fig. 3).

**Fig. 4:**
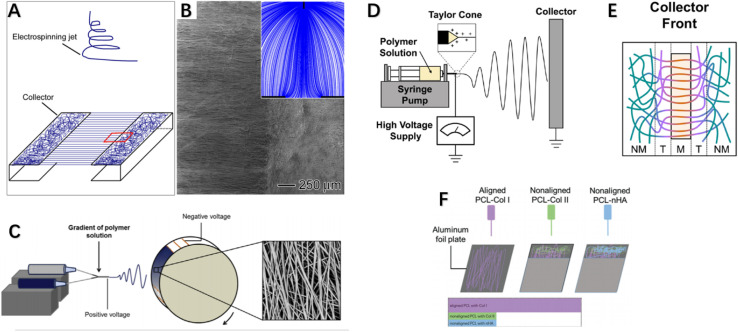
(A) Stapler-like device for manufacturing nanofiber scaffolds and (B) SEM images of randomly arranged and uniaxial nanofiber scaffolds were obtained. Inset: schematic illustration of electric field lines between needle and collector. (C) Electrospinning device with custom rotating collector for fabricating component gradients and SEM images of fibers. (D) Electrospinning apparatus side view, and (E) collector equipped with a single magnet. (F) Schematic illustration of multi-layered electrospinning fiber stacking to achieve alignment-to-random orientation effects.^92–95^

Over the past two decades, electrospinning has been positively explored for fabricating nanofibers, enabling controllable preparation of nanofiber scaffolds with tunable composition, structure, alignment and functionality by modifying material choice, collector design, number of spinnerets, and electrospinning parameters. Typically, aligned nanofibers are produced using a high-speed rotating drum or framed collectors with gap spacing between edges. Researchers have modulated collector design to control electrospun nanofiber structure, rendering them more suitable for repair of highly oriented tissues by achieving directional nanofiber alignment mimicking native microenvironment topography. For example, customized collector configurations such as patterned drums or arrays of conductive nanowires have enabled fabrication of anisotropically aligned nanofiber yarns or woven textiles for tendon/ligament regeneration. Overall, electrospinning offers a versatile nanofibrous scaffold generation platform with tunable structural and biochemical cues to interface tendon/ligament repair. Xie *et al.* developed a collector consisting of two metal frames shaped like staplers, where nanoparticles were deposited in an aligned manner on the metal and a random manner in the gaps,^92^ fabricating an “aligned-to-random” electrospun nanofiber scaffold. Using this scaffold, the tendon–bone insertion site could be modeled as collagen fibers are structured. Studies showed this scaffold exhibited significantly increased modulus and ultimate tensile strength. When cells were cultured on this scaffold, tendon fibroblasts in the aligned and random portions respectively demonstrated highly oriented and non-oriented morphologies, and tendon fibroblasts implanted in the random and aligned portions of the scaffold produced type I collagen rather than type II collagen.^92^ This suggests fibroblasts cultured in this system could generate an appropriate ECM for tendon repair. Fig. 4A and B demonstrates the experimental setup for fabricating a typical “ordered and disordered” electrospun nanofiber scaffold. Kishan *et al.* implemented uniform fibrous mat collection *via* custom collectors equipped with synchronized rotation and utilized periodic copper wires to guide directional fiber alignment (Fig. 4C). Biodegradable polyurethane (BPUR) at varying contents were electrospun to fabricate composition gradient mesh scaffolds with and without fiber orientation for tendon–bone grafting.^93^ The polymer gradient from BPUR50 to BPUR10 along the aligned direction allowed progressive transition in mechanical properties.^93^ In addition to allowing continuous gradient in polymer content, the technique can be used to generate activity gradients along the arrangement direction by adding additives into the polymer solution. Neither the compositional gradients nor fiber alignment of the scaffolds affected cell attachment, and meshes with different alignments exhibited significant stiffness gradients. Both studies employed gap electrospinning for scaffold preparation, requiring almost no additional equipment beyond standard electrospinning apparatus, representing an economical and convenient approach achieving unique microstructural control. However, the method is limited in fabricating scaffolds of large sizes and thicknesses.^96,97^ In this configuration, the ordered and disordered nanofiber regions mimic the graded tendon-to-bone transition. Such modulated nanofiber topography could guide cell behaviors relevant to regenerating the zonal interfaces.

Researchers from Tindell *et al.* demonstrated that magnetically assisted electrospinning allows for fine spatial control over fiber alignments, resulting in wavy interfaces between aligned and random fiber regions.^94^ Ajao *et al.* employed magnetically assisted electrospinning technique using additional cylindrical magnets where well-oriented nanofibers were observed at magnet tops while non-aligned at other positions^98^ (Fig. 4D and E). By adjusting magnet configurations, magnetically assisted electrospinning can achieve various fiber gradients including random, multi-directional and other complex gradients. This method only requires minor modifications to conventional electrospinning setup *via* addition of an array of magnets of different types and configurations to spatially control fiber alignments and generate desired fiber architectures. An advantage of this general device is its ability to be configured in a modular manner without changing the original setup. Researchers also employed auxiliary electrodes to influence electrospinning jet trajectory and thereby control deposition regions and direction of electrospun fibers.^99,100^ Magnetically and electrically assisted fields provide technological basis for fabrication of structurally graded scaffolds needed for tendon–bone interface regeneration. In summary, these studies demonstrated modulated fiber alignments and compositional gradients mimicking tendon–bone transition using simple yet effective electrospinning modifications, holding promise for interface regeneration applications. The techniques of magnetically or electrically assisted electrospinning allow spatial control over fiber deposition enabling generation of fibers with gradients to interface repair. In addition to the aforementioned enhancements made directly on electrospinning equipment, post-processing methods on electrospun fibers can also fabricate scaffolds meeting interface tissue engineering requirements. Manufacturing post-treatment methods can improve orientation by converting poorly aligned fibers into highly oriented meshes *via* combined application of tensile stretching and thermal annealing.^101,102^ Zong *et al.* first described a post-drawing method, investigating the microstructure, morphology and texture of electrospun poly(lactic-*co*-glycolic acid) non-woven membranes after stretching and heat treatment, as well as their degradation and mechanical properties. Results showed the stretched and annealed membranes had higher crystallinity and evident lamellar structuring with improved orientation. Material orientation and tensile strength both increased with rising draw ratios.^103^ Post-drawing provides a viable alternative to obtaining highly aligned fibers or increasing orientation from randomly oriented meshes, with significantly enhanced mechanical properties. However, drawbacks exist such as ∼20% reduction in microstructural porosity post-stretching.^103^ Unlike post-drawing, Yu *et al.* employed photothermal welding technique on aligned fiber scaffolds to successfully fabricate gradients from alignment to randomness.^104^ They premixed ICG directly into the PU solution for electrospinning using a drum-type collector to generate uniaxially aligned nanofibers. Post fabrication, laser irradiation activated the photothermal material (ICG) to generate heat, melting the nanofibers at their melting points and welding them. The photothermally-induced structural changes in nanofiber scaffolds satisfied structural gradation, with subsequent graded mineralization mimicking biological gradients. *In vitro* studies showed the scaffold was biocompatible and guided tendon stem cell morphological elongation and tenogenic and osteogenic differentiation, with *in vivo* investigations *via* immunohistochemical and biomechanical analyses confirming improved rabbit supraspinatus injury healing. To achieve aligned-to-random orientation in electrospun nanofiber membranes, researchers have also employed stacking of multiple electrospun fiber layers to induce orientation transition effects,^95^ as depicted in Fig. 4F where Cong *et al.* separately fabricated membranes including aligned nanofibers PCL (aPCL), random PCL (nPCL), aPCL-collagen I, nPCL-collagen II and nPCL-nanohydroxyapatite (nHA) fibers. They layer-electrospun nPCL-collagen II and nPCL-nHA onto one end of aPCL-collagen I in a stepwise manner. By interlayering Col I, Col II and nHA onto PCL scaffolds, they simulated bone grafts and conducted prosthetic reconstruction. Results showed scaffolds with good biocompatibility, with significantly more neo-fibrocartilage formation observed at experimental group bone–graft junctions *versus* controls, indicating improved bone ingrowth, larger fibrocartilage formation and better biomechanical properties. In addition to the above-mentioned methods of fabricating nanofiber scaffolds with aligned-random orientation gradients *via* various improved apparatus, some researchers have incorporated various active components into conventionally aligned orientation or random orientation fiber membranes to aim at promoting tendon–bone healing. We will discuss these related studies in more detail in the next section (Table 2).

**Table 2:** Summary of methods for fabricating aligned-to-random scaffold

Author	Strategy	Ref.
Xie *et al.*	Combination of gapped electrospinning and conventional electrospinning using a collector composed of two binder clip-shaped metal frames	92
Kishan *et al.*	Fiber alignment achieved by periodic copper wires combined with synchronized rotation of a mesh collector	93
Ajao *et al.*	Addition of auxiliary magnetic field device	98
Tindell *et al.*	Addition of auxiliary magnetic field device	94
Teo *et al.*	Addition of auxiliary electrode device	99
Leon M. *et al.*	Addition of auxiliary electrode device	100
Zong *et al.*	Stretching and thermal treatment	103
Yu *et al.*	Incorporation of photothermal agent into fiber membrane, utilizing photothermal reaction	104
Cong *et al.*	Layering multiple electrospun fiber membranes	95

## Strategies for preparing polymers by electrospinning

**Fig. 5:**
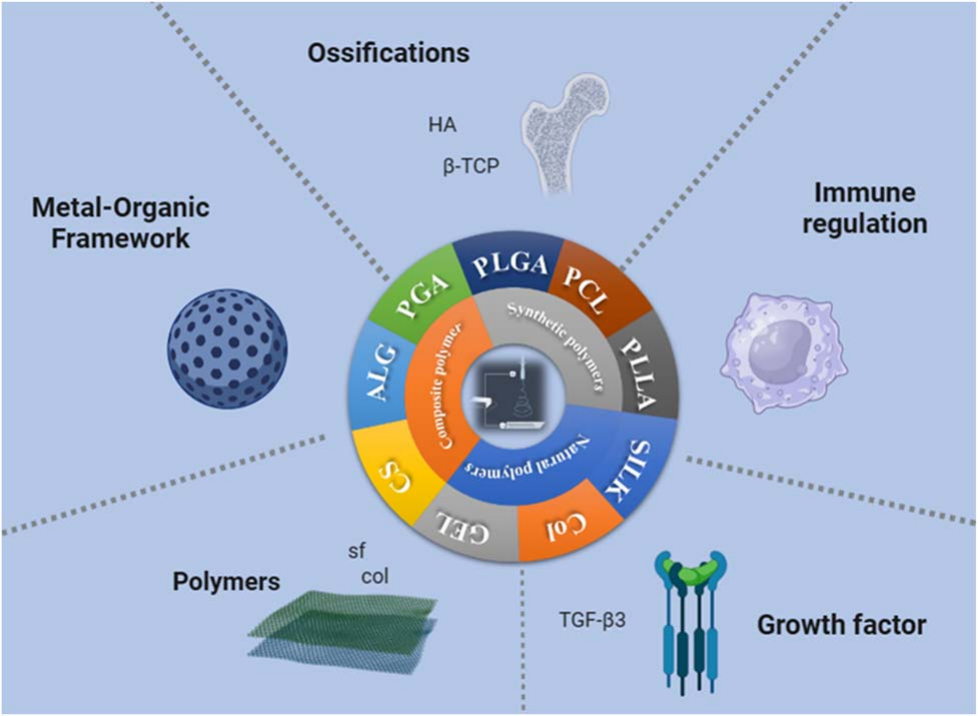
Summary diagram of interface tissue engineering scaffold materials prepared by electrospinning.

The selection of biomaterials plays a crucial role in determining the key parameters of tissue engineering scaffolds, such as structure, biochemical properties, and mechanical performance. However, it is still unclear which biomaterial is the optimal choice for generating such scaffolds. It has been proven that bone-to-tendon tissue engineering scaffolds can be constructed from a variety of biomaterials. Commonly used biodegradable synthetic materials for tissue engineering include poly-l-lactic acid (PLLA),^105^ polyglycolic acid (PGA),^106^ poly(lactic-*co*-glycolic acid) (PLGA),^107,108^ and polycaprolactone (PCL).^109–111^ Because PLGA was approved by the U.S. Food and Drug Administration (FDA), it has gained widespread attention as a scaffold for tissue engineering and drug delivery.^109^ Common natural polymers include alginate,^112^ silk,^107,113,114^ and collagen (Fig. 5).^114–116^

Currently, the primary focus has been on copolymers, blends or polymer-ceramic/bioactive glass composites, aimed at fully leveraging the properties of different materials. Composite strategies attract significant interest in interface tissue engineering as incorporating different materials can better mimic the graded structures of native attachments and exert distinct biological effects. Liu *et al.* employed electrospinning to generate scaffolds with gradient concentration of PLGA and PCL, demonstrating the ability to recreate the tendon–bone microenvironment and reconstruct the interface.^108^ Evaluation of the scaffolds' induction of adipose-derived mesenchymal stem cells (ASCs) osteogenic differentiation showed the gradient in mineral content on the nanofiber surfaces could guide graded ASC differentiation into osteoblasts for rotator cuff repair.^108^ Naghashzargar *et al.* fabricated a novel scaffold material using silk fibroin as the core encapsulated by P3HB and PCL fibers *via* electrospinning. Results indicated this material possessed enhanced tensile strength meeting the mechanical demands for tendon–bone reconstruction, and demonstrated good biocompatibility in L929 cell studies.^117^

Bone loss frequently accompanies tendon–bone injuries, and incorporating bone bioactive materials is a common polymer strategy to enhance electrospun scaffold osteogenic activity. These include calcium phosphate ceramics (particularly hydroxyapatite (HA)^118,119^ and β-tricalcium phosphate (β-TCP)^105,120^), bioactive glasses (mainly silicate-based 45S5 Bioglass®^121,122^). Lv *et al.* incorporated hydroxyapatite (HA) into poly(lactic acid) (PLLA) to fabricate electrospun nanofiber membranes as rotator cuff repair patches. The results showed hydrophilicity of electrospun fibers improved with good cytocompatibility, enhancing alkaline phosphatase (ALP) expression of rat bone marrow mesenchymal stem cells (BMSCs), indicating better induction of rat BMSC osteogenesis.^123^ Soo Kim *et al.* generated a tendon-to-bone structural scaffold consisting of 4 layers – collagen forms the tendon layer, fibrocartilage bonds to chondroitin sulfate to form fibrocartilage, HA forms the mineralized fibrocartilage, and calcium forms the bone layer. Young's modulus increased while elongation decreased.^124^ Further analysis showed that tenocytes, chondrocytes, and osteoblasts throughout the tendon and fibrocartilage layers, as well as osteoblasts in the bone layer, showed enhanced proliferation.^124^ Erisken *et al.*^125^ fabricated β-TCP graded electrospun PCL nanofiber membranes and analyzed their mechanical properties. During tensile deformation, Young's modulus increased gradually along the scaffold with increasing mineral content, whereas elongation decreased.^125,126^ Furthermore, MC3T3 pre-osteoblastic cell attachment and migration showed different behaviors and morphologies along the scaffold.^125,126^ According to these studies, structural and chemical gradients affect mechanical properties and cellular behavior of scaffolds, especially mineral gradients.

Biological factors like TGF-β3 may also be important promotors in the rotator cuff tear healing process. Reifenrath *et al.* studied a TGF-β3 loaded electrospun shellac-grafted-polycaprolactone (CS-g-PCL) fiber scaffold and compared its biomechanical and histological effects on tendon healing with unloaded fiber scaffolds in a rat chronic tendon defect model. The results showed the fiber scaffold with better degradability and biocompatibility, while fibrosis appeared reduced as a foreign body encapsulation and scar formation indicator.^127^ Gao *et al.* synthesized strontium-doped mesoporous bioactive glass nanoparticles (Sr-MBG) *via* a sol–gel method and prepared a biphasic inductive and immunomodulatory electrospun fibrous scaffold containing Sr-MBG (BIIEFS). Mesenchymal stem cells (MSCs) were shown to differentiate osteogenically and chondrogenically when treated with BIIEFS, with multiple bioactive ions being released, and macrophages were confirmed to acquire an M2 phenotype when stimulated with BIIEFS. Experimental results showed that electrospun scaffolds increased the number of M2 macrophages, while synchronous regeneration of tendon, fibrocartilage, and bone was observed, which significantly enhanced supraspinatus tendon-humerus biomechanical strength.^128^

These studies exemplify how incorporating bioactive factors and materials into electrospun scaffolds holds promise for developing clinically translatable therapeutics through regulating the wound microenvironment and cellular responses during interface tissue regeneration. Continued optimizations modulating scaffold composition, topography and biomechanical integrity are important next steps towards achieving functional restoration. Metal ions play important roles in tissue repair and have wide clinical applications. However, their use is limited due to narrow metabolic and healing windows. In this study, Yang *et al.* produced biphasic metal-flexible electrospun fiber membranes from a MOF carrier through continuous electrospinning and matching the longitudinal spatial morphology of multiple tissues simultaneously.^129^ As a carrier, MOF not only enables sustained metal ion release but also promotes osteogenesis and tenogenesis on the scaffold. Studies showed this layered electrospun fibrous structure could accelerate tenogenesis, biological mineralization and vascularization. During *in vivo* validation, the agent was shown to promote tendon and bone tissue repair, as well as fibrocartilage reconstruction, facilitating synchronized regeneration of several tissues at the injured tendon–bone interface. In summary, Using MOFs as the base, electrospun fiber membranes will be biphasic metal-flexible represent a novel class of biodegradable soft scaffolds with tremendous potential for reconstructing tissue defects, especially graded tissue injuries.^129^ These studies exemplify how incorporating bioactive factors and materials into electrospun scaffolds holds promise for developing clinically translatable therapeutics through regulating the wound microenvironment and cellular responses during interface tissue regeneration. Continued optimizations modulating scaffold composition, topography and biomechanical integrity are important next steps towards achieving functional restoration. There is also a kind of acellular matrix materials used as interface tissue engineering to promote tendon and bone healing, but this review mainly focuses on the materials and applications of electrospinning technology, so it will not be discussed (Table 3).

**Table 3:** Summary of electrospun polymer strategies for tendon–bone interface tissue engineering

Author	Polymer strategy	Function	Ref.
Lv *et al.*	PLLA-HA	Induced osteogenic differentiation of BMSCs	123
Liu *et al.*	PLGA-PCL-HA	Induced osteogenic differentiation of ASCs	108
Naghashzargar *et al.*	P3HB-PCL-SF	Met mechanical requirements for tendon–bone reconstruction	117
Soo Kim *et al.*	Collagen-HA	Increased Young's modulus, promoted proliferation of fibroblasts, chondrocytes, and osteoblasts	124
Erisken *et al.*	PCL-β-TCP	Increased Young's modulus, promoted MC3T3 cell adhesion	125 and 126
Reifenrath *et al.*	CS-g-PCL-TGF-β3	Excellent degradation properties, reduced *in vivo* fibrosis	127
Gao *et al.*	BIEFS	Promoted osteogenic and chondrogenic differentiation of MSCs, and polarization of macrophages towards M2 phenotype	128
Yang *et al.*	PLA-HKUST-1/PLA-ZIF-11	Enhanced osteogenesis and tendon formation	129

## Evaluation and analysis of mechanical properties of bionic scaffolds

Since the function of the BTI interface is to bear and transmit loads between mechanically different tissues, it must be able to withstand certain mechanical loads. Evidence also suggests that cells near the interface can sense mechanical forces, which, through genetic regulation, further influence cell migration, extracellular matrix adhesion, and cell proliferation and differentiation.^21^ Therefore, excellent mechanical strength is a necessary condition for the fabricated bionic scaffold. In this section, we will discuss the mechanical strength of some of the electrospun bionic scaffolds mentioned above.

In the study by Xie *et al.*,^92^ tensile tests were used to evaluate the mechanical properties of the scaffold. The scaffold was tested on a tensile testing machine to measure its tensile strength and elastic modulus. Aligned fibers: tensile strength reached approximately 15 MPa, and elastic modulus was 100 MPa. Random fibers: tensile strength was about 5 MPa, and elastic modulus was 50 MPa. The tensile strength of the scaffold depends on the arrangement of the fibers. The tensile strength of the aligned fibers was significantly higher than that of the random fibers. In the study by Kishan *et al.*,^93^ various testing methods were used to evaluate the mechanical properties of the scaffold, including tensile tests, compression tests, and bending tests. The tensile strength of the aligned fiber scaffold was 23.5 ± 1.2 MPa. The tensile strength of the random fiber scaffold was 12.8 ± 0.9 MPa. The elastic modulus of the aligned fiber scaffold was 162 ± 10 MPa. The elastic modulus of the random fiber scaffold was 92 ± 8 MPa. Elongation at break: the elongation at break of the aligned fiber scaffold was 21.7 ± 1.8%. The elongation at break of the random fiber scaffold was 15.3 ± 1.4%. Similarly, the elastic modulus of the aligned fibers was higher than that of the random fibers, indicating that aligned fibers provide stronger mechanical support and stability. Similarly, other studies have recorded tensile strengths ranging from 12 to 45 MPa and elastic moduli from 90 to 220 MPa. Specific data are summarized in Table 4. These studies demonstrate the importance of mechanical properties for tissue engineering scaffolds. In summary, these studies found that aligned fibers generally have superior tensile strength and elastic modulus compared to random fibers. However, considering the complex structural variations of the BTI interface, the quality of a bionic scaffold cannot be judged solely by its mechanical strength.

**Table 4:** Summary of mechanical properties of bionic scaffolds

Tensile strength (aligned)	Tensile strength (random)	Elastic modulus (aligned)	Elastic modulus (random)	Elongation at break (aligned)	Elongation at break (random)	Ref.
15	5	100	50	—	—	92
23.5 ± 1.2	12.8 ± 0.9	162 ± 10	92 ± 8	21.7 ± 1.8	15.3 ± 1.4	93
35	—	200	—	—	—	96
45	—	220	—	—	—	97
30	20	150	100	—	—	94
12	—	90	—	—	—	98
28	—	180	—	—	—	99
18	—	140	—	—	—	100
22	—	160	—	—	—	101
25	—	130	—	—	—	102
20	—	120	—	—	—	103
32	—	210	—	—	—	104
35	—	220	—	—	—	95

## Summary and future perspectives

As part of this article, the author summarizes how electrospinning technology can be used in the design of tendon–bone interface tissues. Various cell types maintain stability within the tendon–bone interface, which enables efficient transmission of muscular forces to the skeleton. The mechanism of tendon–bone interface healing is complex yet crucial for patient prognosis, and current treatments still fall short in achieving complete reconstruction of the tendon–bone interface. The author summarizes the research on the biological and mechanical mechanisms of tendon–bone interface healing and discusses the developmental characteristics and healing mechanisms of injured interfaces, which primarily involve molecular biology, physical factor stimulation, and mechanical stimulation. The article also delves into the strategies and materials involved in scaffold preparation for bone–tendon interface tissue engineering using electrospinning technology. Tissue engineering to achieve regeneration of muscle-tendon/ligament–bone interfaces presents an attractive strategy for providing functional transplants and improving clinical outcomes following injuries. In spite of this, attachment site tissue engineering represents a significant challenge for biologists and engineers because of the complex structure and the critical interdependences between structure, function, and mechanical properties. Tissue engineering has made rapid advances in recent years, but functional attachment site-like tissues have yet to be created. In order to improve the quality of attachment sites, a deeper understanding of their structure–function relationship, as well as mechanisms of attachment site development, homeostasis, and regeneration, is necessary. Biomechanical and biological factors drive the development of naturally occurring insertion sites, which are functionally graded tissues composed of multiple cells types and extracellular matrix components. Transcription factors specific to different types of cells initiate the development of graded interfaces between bone and muscle-tendon or ligament. Consequently, muscle strength signals determine the maturity and growth of the attachment site. As such, fundamental research on the molecular and mechanical factors regulating natural attachment site development and homeostasis is essential for advancing future interface tissue engineering approaches. The interface between soft tissues and bones plays an important role in musculoskeletal function, and its regeneration through tissue engineering promises to improve clinical results. However, there are still several challenges to overcome before the application of current research in clinical practice: (1) regarding the direction of mechanism research, although there have been numerous studies on the molecular mechanisms of tendon–bone interface healing, the core keys to healing remain unclear. (2) In terms of experimental translation, interdisciplinary collaboration is required to demonstrate the classification, composition, and implantation methods of various scaffold materials, aiming to obtain higher-level clinical research and find the most suitable treatment options. (3) Alongside the emergence of new technologies and materials, ethical concerns have also arisen. Complex tissue engineering implants and the introduction of exogenous cells pose risks to patients. Therefore, close attention to ethical issues is still necessary before implementing these treatment strategies.

## Data availability

No data was used for the research described in the article.

## Conflicts of interest

The authors declare that they have no known competing financial interests or personal relationships that could have appeared to influence the work reported in this paper.
